# Intestinal Obstruction due to Bezoar in Elderly Patients: Risk Factors and Treatment Results

**DOI:** 10.1155/2019/3647356

**Published:** 2019-02-17

**Authors:** Fatih Altintoprak, Eyup Gemici, Yasin Alper Yildiz, Mustafa Yener Uzunoglu, Taner Kivilcim

**Affiliations:** ^1^Sakarya University Faculty of Medicine Department of General Surgery, Turkey; ^2^Sakarya University Research and Educational Hospital, Department of General Surgery, Turkey; ^3^Istanbul Bakirkoy Dr. Sadi Konuk Training and Research Hospital, Department of General Surgery, Turkey; ^4^Okan University Faculty of Medicine, Department of General Surgery, Turkey

## Abstract

**Purpose:**

Bezoars are foreign particles from the accumulation of indigestible materials in the gastrointestinal system and a rare cause of mechanical intestinal obstruction. We aimed at investigating differences in risk factors for the development of intestinal obstruction associated with bezoar in elderly patients.

**Methods:**

Hospital records of patients who underwent surgery associated with phytobezoar between January 2004 and May 2016 were retrospectively evaluated. Patients were divided into two groups [<65 years (Group 1) and ≥65 years (Group 2)]. Data were examined regarding presence of comorbidity, history of abdominal surgery, operation time, bezoar site, surgical technique, length of hospitalization, morbidity, and mortality.

**Results:**

Of 121 patients enrolled, 48 (39.7%) were male and 73 (60.3%) were female (range: 24-86 years). Group 1 consisted of 69 patients aged < 65, while Group 2 consisted of 52 patients aged ≥ 65. Comorbidity was reported in 52 (42.9%) patients (mostly diabetes mellitus, 20.7%), while 60 patients (49.6%) had history of abdominal surgery (mostly peptic ulcer, 27.3%). No statistical differences were found between the two groups in terms of sex, bezoar site, surgical technique preferred, history of abdominal surgical intervention, pre- and postoperative CT examination, morbidity rates, and length of hospitalization. But, ratio of peptic ulcer operations history, presence of total comorbidity, and time of surgery decision was higher in Group 2 patients.

**Conclusion:**

In bezoar-related intestinal obstruction, duration and outcome of treatment are not affected by age distribution. Possibility of bezoar should primarily be considered in elderly patients with history of peptic ulcer operation.

## 1. Introduction

Acute mechanical intestinal obstruction (AMIO) is a condition in which the contents of the intestinal lumen are prevented from advancing due to various causes and that generally requires emergency surgical intervention. Although the etiology of intestinal obstruction is varied, this condition, which constitutes approximately 20% of all emergency surgical interventions, is mostly caused by adhesions associated with previous abdominal surgical interventions [1]. Notwithstanding the fact that adhesion represents the most encountered etiology in all age groups, malignancy as a prediagnosis should primarily be considered and excluded during diagnosis in the elderly population [2].

Bezoar is a term used to describe indigestible materials that are orally ingested and that accumulate in the gastrointestinal system as intraluminal foreign particles. They have various names according to the material they are composed of, including phytobezoars, trichobezoars, and lactobezoars. The most encountered form is the phytobezoar, which is associated with the consumption of fibrous food [3]. Studies have demonstrated that phytobezoars are generally one of the least common and rare causes of intestinal obstruction [4]. However, they are the most commonly reported etiological factor of intestinal obstruction in certain geographical areas where very high fiber-containing food (e.g., persimmons) are grown and consumed [5]. Apart from fiber-rich food consumption, many risk factors have also been described that facilitate the development of bezoars, including diabetes mellitus, history of ulcer surgery, hypothyroidism, and dental problems in the elderly as a result of the inability to chew food properly [6, 7].

In this study, we evaluated the results of patients who underwent surgical intervention due to intestinal obstruction associated with phytobezoars. The differences in the risk factors for the development of intestinal obstruction associated with the bezoar site, surgical treatment results, and bezoars were investigated in patients < 65 years and in those ≥ 65 years old.

## 2. Methods

Hospital record files of patients who underwent surgical treatment due to AMIO associated with phytobezoars at the General Surgery Department of Sakarya University Research and Educational Hospital, between January 2004 and May 2016, were retrospectively evaluated. The patients were divided into two groups [< 65 years (Group 1) and ≥ 65 years (Group 2)] to determine the differences between two groups. The patient files from both groups were examined with respect to demographic data, presence of comorbidity (risk factors for surgical intervention such as chronic diseases), history of abdominal surgical intervention, operation time, bezoar site, surgical technique used, length of hospital stay, and morbidity and mortality rates.

Following upper gastrointestinal (GI) endoscopic examination in patients diagnosed with gastric phytobezoars, seven patients who received nonsurgical treatment, 14 patients who underwent elective surgery from the diagnosis of phytobezoars with gastric localization, 2 patients who underwent surgery and were diagnosed with trichobezoars, and 1 patient diagnosed with colonic bezoar were excluded from the study. Also, when comparison was made between the morbidity-mortality rates and the surgical technique, patients (n = 4) who underwent segmental small intestinal resection following the diagnosis of local ischemia and/or necrosis of any site in the small intestine associated with bezoar pressure were excluded from the study.

All patients were subjected to gastrostomy (surgical removal of the bezoar through an opening in the gastric lumen) following the diagnosis of a second bezoar, apart from the bezoar that caused small intestinal obstruction during the operation.

The Number Cruncher Statistical System (NCSS) 2007 program (Kaysville, Utah, USA) was used for all statistical analyses. Descriptive statistical methods (mean, standard deviation, median, frequency, ratio, minimum, and maximum) were used to evaluate the study data. The Mann-Whitney U-test was used to compare parameters showing abnormal distribution between the two groups. A comparison of qualitative data was made using the Pearson's chi-square test, Fisher's exact test, the Fisher-Freeman-Halton test, or Yates' Continuity Correction test (Yates' corrected chi-square). Logistic regression analysis was used to evaluate the risk factors affecting age during multivariate evaluation. P-values < 0.01 and < 0.05 were considered to indicate statistical significance.

## 3. Results

Of the 121 patients who were enrolled in the study, 48 (39.7%) were male and 73 (60.3%) were female (range: 24-86 years; mean: 59.0 ± 13.69 years). Comorbidity was reported in 52 (42.9%) patients (most commonly diabetes mellitus, 20.7%), while 60 patients (49.6%) had a history of abdominal surgical intervention from various causes (most commonly from peptic ulcer, 27.3%). No hypothyroidism was detected in any of our patients. Since this study has retrospective nature and chewing problems caused by dental issues are not routinely questioned during hospital admission, we could not evaluate this parameter. Patients' comorbidity details are shown in Table 1.

Surgery was performed within a period of 1–11 days (1.80 ± 1.84) depending on the clinical findings at consultation, radiological findings, need for preoperative medical treatment, necessity for consultation related to comorbidity, and parameters required for postoperative intensive care follow-up.

**Figure 1:**
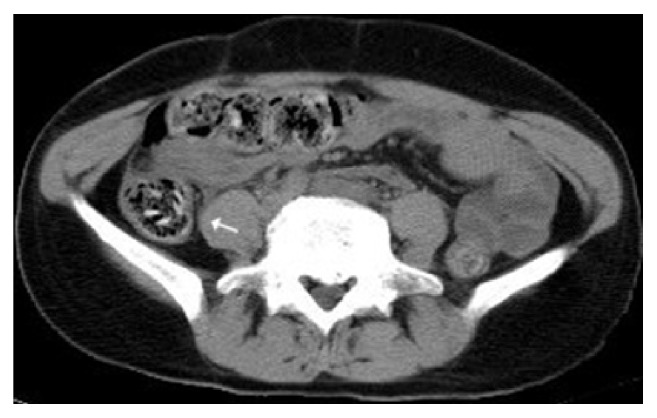
CT imaging; intraluminal phytobezoar appearance with persimmon seeds (arrow).

The presence of a bezoar and its site was determined with abdominal computed tomography (CT) in 89 patients (73.5%) at least once during the preoperative period (Figure 1). On the other hand, the decision for surgery was made in 32 patients (26.4%) without preknowledge of the presence of a bezoar following a series of abdominal examinations, leukocyte count follow-up, and conventional abdominal x-ray examination. The diagnosis of intestinal phytobezoar was confirmed with surgical findings in all patients.

Evaluation of the bezoar site determined during surgery demonstrated the presence of a bezoar in the duodenum in two patients (1.6%), in the jejunum in 38 patients (31.4%), in the ileum in 38 patients (31.4%), in the stomach and jejunum in 14 patients (11.5%), and in the stomach and ileum in 29 patients (23.9%).

**Figure 2:**
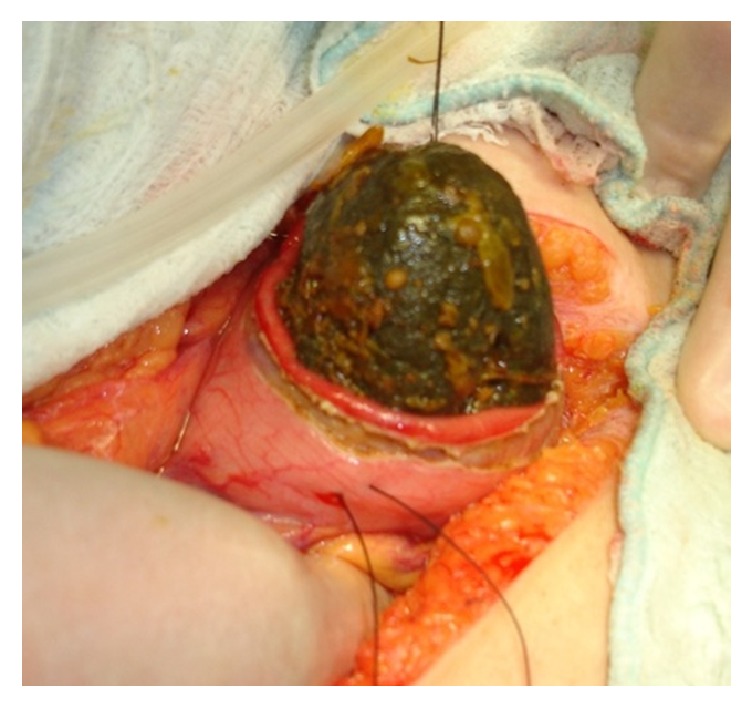
Intraoperative view; removing phytobezoar with gastrotomy.

The most practiced surgical technique reported was milking (fragmentation of the obstruction-causing bezoar in the lumen with the hand and then pushing it forward into the colon; 45 patients, 37.1%). Other commonly used techniques were enterotomy (incision into the intestine at the obstruction site and removal of the bezoar; 36 patients, 29.7%), gastrostomy (Figure 2) + milking (25 patients, 20.6%), and gastrostomy + enterotomy (15 patients, 12.3%). (Gastrotomy procedure was performed in patients with synchronized bezoar detected in the stomach.)

The period of hospitalization ranged between 2 and 52 days (mean: 7.45 ± 6.17 days). Various morbidities were reported in 24 patients (19.8%) during the postoperative period, the most common of which was wound infection (17 patients, 14.0%). Three patients (2.4%) died after being followed up in the intensive care unit during the postoperative period.

All patients whose treatment period ended with mortality were in Group 2; in all cases, there was a history of abdominal surgical intervention and an inability to perform abdominal CT due to various reasons (e.g., contrast allergy and technical reasons).

The patients' demographic data, bezoar site, surgical techniques, and morbidity-mortality rates are shown in Table 2.

Group 1 consisted of 69 patients aged < 65, while Group 2 consisted of 52 patients aged ≥ 65.

No statistical differences were found between the two groups in terms of sex, history of abdominal surgical intervention, pre- and postoperative CT examination, morbidity rates, and length of hospitalization (p > 0.05 for all parameters) (Table 3).

**Figure 3:**
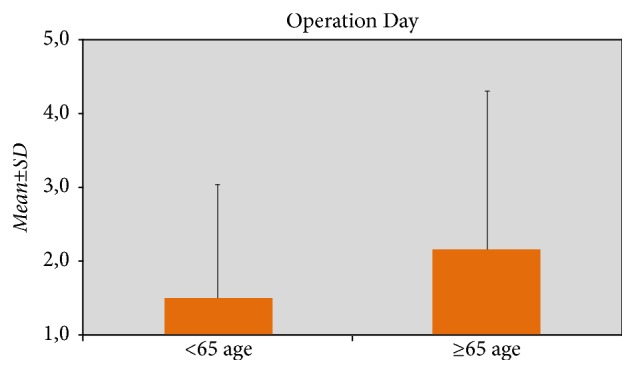
Days scheduled for operation according to ages.

The parameters that were found to be statistically significant are as follows:

- From past abdominal operations, the ratio of peptic ulcer operations was higher in Group 2 (p = 0.003; p < 0.01).
- The presence of total comorbidity was higher in Group 2 (p = 0.001; p < 0.01).
- With regard to comorbidity, the prevalence of diabetes mellitus was higher in Group 2 (p = 0.001; p < 0.01).
- The time of surgery decision in Group 2 patients was longer compared to Group 1 patients (p = 0.015; p < 0.05) (Figure 3).

Statistical comparisons between the groups are shown in Table 3.

The effect of history of past peptic ulcer surgery and the presence of diabetes mellitus were evaluated using logistic regression analysis to determine the effect of risk factors that affect patients ≥ 65 years of age, and the model was significant and the coefficient of expression (72.7%) was on a good level. The odds ratio for the history of past peptic ulcer surgery was 3.635 (95% confidence interval (CI): 1.437–9.200), whereas that for diabetes mellitus was 7.454 (95% CI: 2.498–22.244) (Table 4).

## 4. Discussion

Intestinal obstruction usually involves the small intestine, and the most common etiology worldwide is adhesion that develops following abdominal surgical interventions [1, 8]. Bezoars are rarely encountered and are responsible for only 4.5% of intestinal obstructions. However, the rate of intestinal obstructions is very high (60%) in patients with bezoars [9]. Malignancy usually comes to mind when encountering AMIO in the elderly and measures to confirm these diagnoses are often investigated. In this study, it is emphasized that the presence of bezoar possibility in elderly patients may be more than expected if there are some factors, such as diabetes mellitus and previous peptic ulcer surgery history.

Persimmon is a fruit that grows endemically in certain geographical areas and has been associated with the development of bezoars in many studies [10]. AMIO cases associated with phytobezoars are commonly encountered in our region, which is a natural habitat for the growth of persimmon. Postpeptic ulcer surgery complications such as abnormalities in gastric motility, hypoacidity, pyloric function loss, and the creation of a wide gastric outlet have been associated with the development of bezoars and bezoar-related AMIO [11–14]. Peptic ulcer operation history rate was high in our study (in about 1/4 of our patients), which is consistent with the literature; moreover, this rate was higher in patients with a history of abdominal surgical intervention (in about 1/2). Similarly, the presence of certain diseases (myotonic dystrophy, hypothyroidism, and diabetes mellitus) that cause a decrease in gastric and/or whole gastrointestinal system motility has been described as a risk factor for the development of bezoars [15–17]. Diabetes mellitus was found in 20% of our patients and was the most common disease among all comorbidities (48%).

The clinical picture and the laboratory and conventional abdominal x-ray findings of intestinal obstructions associated with bezoars are not very different from those of other causes of obstruction. Abdominal CT imaging provides valuable information to determine the level of obstruction and any etiological factors involved [18, 19]. Abdominal CT has an additional advantage with regard to determining whether synchronized bezoars are present on dilated intestinal segments for bezoar types with specific appearances proximal to the level of the obstruction [13]. Preoperative detection of the presence and site of a synchronized bezoar is important for optimizing the surgical strategy. Previous studies have reported cases where repeated surgery was necessary because of nondetected synchronized bezoars or in cases overlooked on CT examination [20]. The preoperative diagnosis and site detection of bezoars by abdominal CT were made in the majority of our patients (73.6%). Thus, gastrotomy could be planned in preoperative period in patients who were found to be synchronized bezoar in the stomach.

Bezoars most often occur in the stomach [21]. Although small bezoars may leave the stomach through the pyloric opening, large bezoars may only be removed through the small intestine in patients where wider gastric routes are provided (gastrojejunostomy and pyloroplasty). However, it is suggested that bezoars may also develop and grow in the small intestine in cases where pathology (stricture or diverticulitis) obstructs passage in the lumen [22]. The narrowest sites in the gastrointestinal system that a swallowed object may reach are the pylorus and the ileocecal region. It is hence normal to detect obstructions associated with bezoars in these areas. No obstructions are generally observed in the stomach apart from those related with diseases that cause narrowing of the pylorus since the stomach produces powerful contractions due to its large volume and thick muscle layers. In our study, gastric bezoar rate was %35; however, all bezoars responsible for obstruction were in the small intestine and, in these cases, gastric bezoars were only secondary. Furthermore, bezoars in our study were not only located at the narrowest part of the small intestine but also caused obstructions in other intestinal segments, including the duodenum.

It is very important to exclude the possibility of bezoars in patients who present with signs of small intestinal obstruction and in those with a history of abdominal surgical intervention. This is due to the fact that obstruction in these patients is most commonly due to adhesions, and nonoperative treatment options are commonly considered [23, 24]. However, surgical treatment is inevitable for intestinal obstructions associated with bezoars. Since comorbidity is more common in elderly patients, it is important to detect bezoars that require surgical intervention early to prevent loss of time and reduce morbidity-mortality rates to acceptable levels [25]. Consequently, abdominal CT evaluation in this group of patients should be considered at an early stage. In our study, some patients died at the end of the treatment period, particularly those with a history of abdominal surgical intervention and those who received a prediagnosis of adhesion and who had to receive surgical treatment following failure of medical treatment.

A search of the literature using keywords such as bezoar and intestinal obstruction produced many studies. However, we found no study that investigated differences between age groups or that studied elderly patients specifically. In our study, no differences were observed between patients < 65 years and those ≥ 65 years of age in terms of gender, bezoar site, preferred surgical techniques, history of abdominal surgical intervention, morbidity rates, and length of hospitalization. We demonstrated that the presence of intestinal obstructions associated with bezoars did not affect the treatment period or results according to age group and that it was more important to make treatment plans after considering the diagnosis of bezoar.

Total comorbidity and operation times were significantly higher in Group 2 patients. This result is thought to be normal since comorbidity increases with age, and the operation period (completion of necessary preoperative consultation, evaluation of biochemistry parameters, and preparation of conditions in the intensive care unit) for these patients is known to be longer [26].

Two important results of our study were that peptic ulcer surgery emerged as the most commonly performed type of abdominal surgical intervention and that diabetes mellitus was the most encountered comorbidity, with rates significantly higher in patients ≥ 65 years. These two parameters did not show statistical differences between the two age groups in terms of the treatment period. The most important and effective difference was found to be in diagnosing bezoars on the very first visit. From our experience, doing so would greatly speed up appropriate treatment planning, thereby increasing the possibility of a better outcome. It is important to stress that a history of peptic ulcer surgical operation is more important between these two parameters when considering treatment, since diabetes mellitus is a very common occurrence in the elderly age group. Moreover, advances in medical treatment within the last 20 years have led to the exclusion of elective peptic ulcer surgical operations from routine surgical protocol. As a result, a history of peptic ulcer surgery is most commonly encountered in the elderly patient population [27].

In conclusion, intestinal obstructions are frequently encountered in geographical regions endemic in phytobezoars, which are the more common etiological factor. A bezoar diagnosis should primarily be considered, especially in elderly patients who present with a clinical picture of intestinal obstruction and a history of peptic ulcer operation.

## Figures and Tables

**Table 1:** Coincidental diseases in all patients.

Demographic findings (n=121)	n	%
Coincidental disease^1^	52	43.0
(i) Diabetes Mellitus	25	20.7
(ii) Hypertension	19	15.7
(iii) Incisional hernia	5	4.1
(iv) Prostate diseases (benign prostate hyperplasia)	5	4.1
(v) Cardiac and coronary artery diseases	3	2.5
(vi) Arrhythmia	3	2.4
(vii) Chronic pulmonary diseases	3	2.5
(viii) Cerebro-vascular diseases	2	1.6
(ix) Renal diseases (chronic renal failure)	1	0.8
(x) Psychiatric disorder	1	0.8
(xi) Psoriasis	1	0.8
(xii) Obesity	1	0.8

^1^Eleven patients had two or more diseases.

**Table 2:** Demographic data, bezoar sites, surgical techniques, and morbidity-mortality rates of all patients.

		Min-Max (Median)	Mean ± SD
Age *(years)*		24-86 (61)	59.0±13.69
Operation Time *(days)*		1-11 (1)	1.80±1.84
Length of hospitalization *(days)*		2-52 (6)	7.45±6.17

		n	%

Sex	Male	48	39.7
	Female	73	60,3

Surgical technique	Milking	45	37.1
	Enterotomy	36	29.7
	Gastrostomy+Milking	25	20.6
	Gastrostomy+Enterotomy	15	12.3

Bezoar site	Duodenum	2	1.6
	Jejunum	38	31.4
	ileum	38	31.4
	Stomach + Jejunum	14	11.5
	Stomach + İleum	29	23.9

History of abdominal		60	49.6

surgical intervention	

History of peptic ulcer surgery		33	27.3

Total comorbidities		52	42.9

Presence of diabetes mellitus		25	20.7

Morbidity		24	19.8

Mortality		3	2.4

Abdominal CT examination		89	73.6

**Table 3:** Descriptive and comparative properties according to age.

		Age		*p*
		<65 years	≥65 years	
		(n=69)	(n=52)	
Sex	*Male*	27 (39.1)	21 (40.4)	^*a*^ *1.000*
	*Female*	42 (60.9)	31 (59.6)	

History of abdominal surgical intervention		33 (47.8)	27 (51.9)	^*c*^ *0.655*

History of peptic ulcer surgery		11 (15.9)	22 (42.3)	^*a*^ *0.003* ^*∗∗*^

Total comorbidity		17 (24.6)	35 (67.3)	^*a*^ *0.001* ^*∗∗*^

Diabetes mellitus		7 (10.1)	18 (34.6)	^*a*^ *0.001* ^*∗∗*^

Morbidity		12 (17.4)	12 (23.1)	^*a*^ *0.585*

Abdominal CT Examination		52 (75.4)	37 (71.2)	^*a*^ *0.755*

Operation time (days)	*Mean ± SD*	1.52±1.53	2.17±2.14	^*d*^ *0.015* ^*∗*^
	*Min-Max (Median)*	1-11 (1)	1-11 (1)	

Length of Hospitalization (days)	*Mean ± SD*	6.58±4.07	8.61±8.07	^*d*^ *0.104*
	*Min-Max (Median)*	2-24 (5)	2-52 (7)	

^*a*^ *Yates' Continuity Correction.*

^*c*^ *Pearson'sChi-Square Test.*

^*d*^ *Mann-Whitney U-Test.*

^*e*^ *Fisher-Freeman-Halton Test.*

^*∗∗*^*p<0.01*

^*∗*^*p<0.05*

**Table 4:** Logistic regression analysis of risk factors effective in patients ≥65 years.

	P	Odds ratio	95% CI	
			Lower	Upper
History of peptic ulcer surgery	0.006^*∗*^	3.635	1.437	9.200
Diabetes mellitus	0.0001^*∗*^	7.454	2.498	22.244

^*∗*^*p<0.01*

## Data Availability

The data used to support the findings of this study are included within the article.
