# Corrigendum to “Intestinal Obstruction due to Bezoar in Elderly Patients: Risk Factors and Treatment Results”

**DOI:** 10.1155/2019/1568134

**Published:** 2019-09-02

**Authors:** Fatih Altintoprak, Eyup Gemici, Yasin Alper Yildiz, Mustafa Yener Uzunoglu, Taner Kivilcim

**Affiliations:** ^1^Sakarya University, Faculty of Medicine, Department of General Surgery, Turkey; ^2^Sakarya University, Research and Educational Hospital, Department of General Surgery, Turkey; ^3^Istanbul Bakirkoy Dr. Sadi Konuk Training and Research Hospital, Department of General Surgery, Turkey; ^4^Okan University, Faculty of Medicine, Department of General Surgery, Turkey

In the article titled “Intestinal Obstruction due to Bezoar in Elderly Patients: Risk Factors and Treatment Results” [1], Drs. Yasin Alper Yildiz and Mustafa Yener Uzunoglu were mistakenly linked to the third affiliation. They should have been linked to the second affiliation. The updated authors' list and affiliations' list are shown above.
